# Optimal diagnostic fever thresholds using non-contact infrared thermometers under COVID-19

**DOI:** 10.3389/fpubh.2022.985553

**Published:** 2022-11-24

**Authors:** Fan Lai, Xin Li, Tianjiao Liu, Xin Wang, Qi Wang, Shan Chen, Sumei Wei, Ying Xiong, Qiannan Hou, Xiaoyan Zeng, Yang Yang, Yalan Li, Yonghong Lin, Xiao Yang

**Affiliations:** ^1^Obstetrics Department, Chengdu Women's and Children's Central Hospital, School of Medicine, University of Electronic Science and Technology of China, Chengdu, China; ^2^Psychiatry Department, The Fourth People's Hospital of Chengdu, School of Medicine, University of Electronic Science and Technology of China, Chengdu, China

**Keywords:** coronavirus, epidemiology, body temperature (BT), mass screening, COVID-19

## Abstract

Fever screening is an effective method to detect infectors associated with different variants of coronavirus disease 2019 (COVID-19) based on the fact that most infectors with COVID-19 have fever symptoms. Non-contact infrared thermometers (NCITs) are widely used in fever screening. Nevertheless, authoritative data is lacking in defining “fever” at different body surface sites when using NCITs. The purpose of this study was to determine the optimal diagnostic threshold for fever screening using NICTs at different body surface sites, to improve the accuracy of fever screening and provide theoretical reference for healthcare policy. Participants (*n* = 1860) who were outpatients or emergency patients at Chengdu Women's and Children's Central Hospital were recruited for this prospective investigation from March 1 to June 30, 2021. NCITs and mercury axillary thermometers were used to measure neck, temple, forehead and wrist temperatures of all participants. Receiver operating characteristic curves were used to reflect the accuracy of NCITs. Linear correlation analysis was used to show the effect of age on body temperature. Multilinear regression analysis was used to explore the association between non-febrile participant's covariates and neck temperature. The mean age of participants was 3.45 ± 2.85 years for children and 28.56 ± 7.25 years for adults. In addition 1,304 (70.1%) participants were children (≤12), and 683 (36.7%) were male. The neck temperature exhibited the highest accuracy among the four sites. Further the optimal fever diagnostic thresholds of NCITs at the four body surface measurement sites were neck (36.75 °C, sensitivity: 0.993, specificity: 0.858); temple (36.55 °C, sensitivity: 0.974, specificity: 0.874); forehead (36.45 °C, sensitivity: 0.961, specificity: 0.813); and wrist (36.15 °C, sensitivity: 0.951, specificity: 0.434). Based on the findings of our study, we recommend 36.15, 36.45, 36.55, and 36.75 °C as the diagnostic thresholds of fever at the wrist, forehead, temple and neck, respectively. Among the four surface sites, neck temperature exhibited the highest accuracy.

## Introduction

Since the first case of coronavirus disease 2019 (COVID-19) was diagnosed in December 2019, the COVID-19 pandemic has evolved with the appearance of several variants (Alpha, Beta, Gamma, Delta and Omicron) (1–6) which affected billions of people around the world and became a significant global public health crisis.

Considering that, unlike previous strikes of COVID-19, the current epidemic is small-scale and cannot be eliminated in a short time, it is not feasible to adopt severe measures such as nationwide nucleic acid measurement and city-wide blockades (7–10). To identify and exclude close contacts of COVID-19 infectors is a difficult but necessary challenge that must be undertaken. Too strict COVID-19 management policy will lead to stagnation or even retrogression of social and economic development (11). Screening out the possible COVID-19 infectors without affecting the social order is conducive to the simultaneous development of economy while ensuring people's health (12, 13). Fever screening is an effective and low-cost method to detect infectors associated with different variants of COVID-19 based on the fact that most infectors with COVID-19 have fever symptoms (14–17). Among 55,924 laboratory confirmed cases of COVID-19 in China, 87.9% had a “fever” (no cut-off point reported). Therefore, the WHO announced that temperature screening was sufficient to detect the majority of COVID-19 cases, and recommended that fever screening checkpoints be positioned at the entrance of public places for screening out potential COVID-19 infectors (18).

Rapid testing, accuracy, and convenience are the key features required for fever screening (19, 20). Compared with mercury axillary thermometers (MATs), non-contact infrared thermometers (NCITs) are more suitable for fever screening because of the rapidity and convenience (21, 22). Nevertheless, authoritative data is lacking in defining “fever” at different body surface sites when using NCITs. Previous studies indicated that the temperature measurements of NCITs were influenced by body surface measurement sites, ambient temperatures and instruments (23–26). Meanwhile, regarding differences in religious beliefs and modes of dressing habits, as well as certain personal factors (local trauma or disability, for example), it is essential to determine the optimal diagnostic fever thresholds at different body surface measurement sites.

In our previous preliminary study (27), we have calculated the theoretical optimal diagnostic threshold (the maximal sum of sensitivity and specificity) at each body surface site. For instance, when 37.4 °C at neck was used as fever threshold, the sensitivity of fever screening was 0.866 while the specificity was 0.846. However, a sensitivity of 0.866 does not meet the criteria for fever screening. In the actual fever screening, we tend to reduce the fever threshold to improve the sensitivity. Meanwhile, for advanced age and frailty can blunt febrile responses to illness, the temperature used to define fever can influence the clinical recognition of COVID-19 symptoms. A lower fever threshold would increase the number of residents recognized as having symptomatic infection, potentially leading to earlier detection and more rapid implementation of therapeutic interventions and infection prevention and control measures (28, 29).

Hence, in this study, we attempted to ascertain the optimal diagnostic threshold for fever screening using NICTs at different body surface sites, unify fever reference standards for improving accuracy and standardization of fever screening under COVID-19.

## Materials and methods

### Sample and data

This study included outpatient and emergency participants willing to participate from March 1 to June 30, 2022. To explore the optimal diagnostic threshold of fever clearly, the participants in this study were not restricted in sex and age (from the newborn to the elderly). The participants with severe acute disease who required rescue, those with physical disability/disorder or mental illness, and individuals with physical cooling or antipyretic paste covering were excluded from the study. The study has been approved by the Ethics Committee of the Chengdu Women's and Children's Central Hospital (No. 202040) and registered in Chinese Clinical Trial Registry (No. ChiCTR2100049560). Written informed consents have been obtained from all participants.

Standardized questionnaires (Supplementary Table 1) were used to collect the sociodemographic data of participants. The data regarding participants' preference for body surface sites for fever screening were obtained from the questionnaires. For children unable express their wishes, their parents/guardians completed the questionnaires on their behalf. All body temperature data were measured using MATs (Model number: CRW-11, accuracy ± 0.1 °C, accuracy range 35.0–42.0 °C, Guangzhou Weierkang Medical Devices Co., Ltd) and NCITs (Model number: JXB-178, accuracy ± 0.2 °C, accuracy range 32.0–42.0 °C, Guangzhou Weierkang Medical Devices Co., Ltd). All body temperature data shall be recorded instantly after measurement. All NCITs and MATs were calibrated before use. Ambient temperature and humidity measuring instrument (Model number: WS2021, accuracy ± 1.0 °C, temperature range 0–45.0 °C, humidity range 0–95 %RH, Tianjin Kehui Instrument Factory, China) was used to measure the ambient temperature and humidity.

### Measures of variables

All investigators have mastered standardized operating procedures of NCITs and MATs (including body surface measurement sites and distance away from surface, ambient temperature measurement and data input) (27). The fever screening point is located at the entrance of the hospital, and the parking lot is outside the hospital. Participants arriving by various modes of transportation (walking, public transport or driving) all need to walk 400 meters to the entrance of the hospital.

All NCITs and MATs were calibrated before use. Wrist, neck, temporal and forehead temperatures were measured using the identical company and the same model of NCITs, 5 cm away from the body surface. MATs were used to detect axillary temperature, after swing the thermometer < 35 °C and wiping the sweat from the armpit the MAT was placed under the armpit for more than 10 min. Although some studies have shown that the accuracy of axillary temperature measured by MATs is not the highest, it was still used as a reference standard to analyze accuracy of NCITs at the four body surface sites in our study based on the following reasons: First, the axillary temperature measured by MATs is relatively stable, while NCITs may vary according to ambient temperature, measurement distance, model and manufacturer; Second, nowadays, MATs are still the most commonly thermometer in poor and developing regions and countries due to its portability and inexpensiveness. Third, under COVID-19, oral temperature measurement may increase the risk of infection, and anal temperature is not suitable for large-scale screening, so they are not suitable as fever screening standard (31, 57). Fever was diagnosed when axillary temperature measured by MATs ≥ 37.3°C (32).

### Models and data analysis procedure

Receiver operating characteristic curve (ROC curve) is a tool used to describe the discrimination accuracy of a diagnostic test or prediction model (58), which can illustrate the diagnostic ability of a binary classifier system as its discrimination threshold is varied. The area under the ROC curve (AUC) is a measure of how well a parameter can distinguish between two diagnostic groups (fever/normal), the closer the AUC is to 1, the higher the efficiency of fever screening. In our study, the cut-off values are fever diagnostic thresholds, fever was diagnosed when body temperature measurement exceeded cut-off value. Sensitivity is the probability that participants with fever will be correctly identified, specificity is the probability that non-febrile participants will be correctly excluded. The Youden indexes (sum of sensitivity and specificity minus 1), were used to reflect the accuracy of fever screening of different body surface sites and different fever diagnostic thresholds.

SPSS version 25.0 software (IBM, Armonk, NY, USA) was used for all statistical analyses. Data were dealt with independent *t*-test and described as mean ± standard deviation if variables were in accordance with a normal distribution. Otherwise, variables were described as median ± quartile and examined by the Kruskal–Wallis test. ROC curves were used to reflect the accuracy of NCITs at different body surface sites in different groups. Multivariable linear regression analysis was used to detect the associations between non-febrile participant's covariates and neck temperature. Variance inflation factor (VIF) is a measure of the amount of multicollinearity in a set of multiple regression variables. A high VIF (> 10) indicates that the associated independent variable is highly collinear with the other variables in the model and need to be deleted. Covariates were selected according to the difference variables in univariate analysis and the factors reported in previous studies that would affect body temperature. The equation of linear regression line was further used to analyze the correlation between body temperature and age in non-febrile participants. All statistical tests were two-sided, and *P* < 0.05 indicated statistical significance, unless otherwise stated.

## Results

The study initially recruited 2,013 participants. However, 153 participants who withdrew or whose data was completely lost were excluded. The final analysis included 1,860 participants. The participants' characteristics are summarized in Table 1. The mean age of participants was 3.45 ± 2.85 years for children and 28.56 ± 7.25 years for adults. In addition 1,304 (70.1%) participants were children (≤12), and 683 (36.7%) were male because we are a specialized hospital for women and children. Among the participants, 823 (44.2%) were tentatively diagnosed with fever based on axillary temperature measured by MATs ≥ 37.3°C. Of these, 691 (84.0%) were children and 132 (16.0%) were teens and adults. The humidity ranged in 30–90 %RH (mean: 58.30 ± 12.90) and ambient temperature ranged in 9°–32°C (mean: 22.5°C ± 4.09 °C).

**Table 1:** Description of the participant's characteristics.

**Variables**	**Total = 1860**
Sex (male)	683 (36.7%)
Age (year)	
Children (≤12 y)	3.45 ± 2.85
Teens and adults (>13 y)	28.56 ± 7.25
Participant types	
Children (≤12 y)	1304 (70.1%)
Boys	631 (48.4%)
Girls	673 (51.6%)
Teens and adults (>13 y)	556 (29.9%)
Males	52 (7.2%)
Pregnant women	186 (33.5%)
Non-pregnant women	318 (57.3%)
Fever with MATs	823 (44.2%)
Children (≤12 y)	691 (84.0%)
Teens and adults (>13 y)	132 (16.0%)
Body temperature data	
Participant types (Axillary temperature)	
Boys	37.33 ± 0.94
Girls	37.38 ± 0.98
Males (>13 y)	37.10 ± 0.92
Pregnant women	37.21 ± 0.99
Non-pregnant women	37.07 ± 0.90
Surface sites	
Forehead temperature	36.87 ± 0.84
Temple temperature	36.91 ± 0.87
Neck temperature	37.48 ± 0.92
Wrist temperature	36.70 ± 0.91
Axillary temperature	37.36 ± 0.86

Temperatures at the wrist, forehead, temple and neck measured by NCITs and axillary temperatures measured by MATs were compared. The results revealed that the neck temperature was closest to the axillary temperature, whereas the wrist, forehead and temple temperatures lower than that (Table 1). Among several major participant populations, the average body temperature in children was higher than that of teens and adults (*p* < 0.001), and that of pregnant women was slightly higher than that of non-pregnant women (*p* = 0.027) (**Figure 2A**); the temperature of females was slightly higher than that of males, but with no statistical difference.

**Figure 1:**
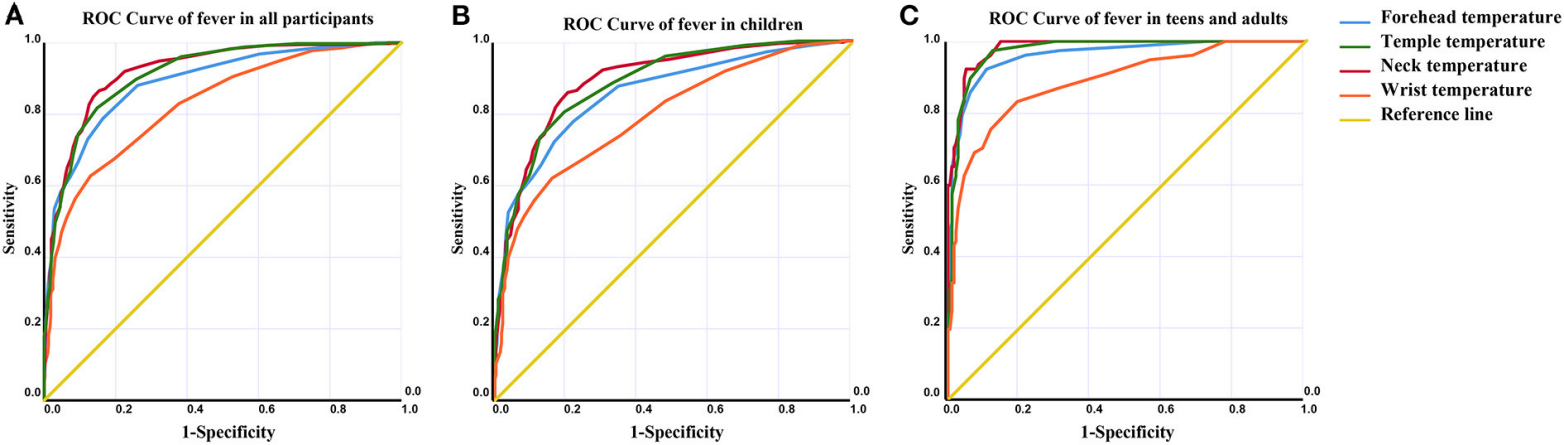
ROC curves were used to reflect the accuracy of NCITs at different body surface sites in different groups. **(A–C)** When axillary temperature ≥ 37.3 °C was used as the diagnostic criterion of fever, ROC curves of fever in all participants, children, and teens and adults, respectively were shown.

Axillary temperature of ≥ 37.3 °C was used as the diagnostic criterion of fever, ROC curves were used to reflect the accuracy of NCITs at different body surface sites in different groups (Figure 1). Surprisingly, when all participants were involved in the ROC curve, the maximum AUC was 0.922 among the four body surface sites. The maximum specificity was only 0.623 when the sensitivity fulfilled the requirements of fever screening (single sensitivity > 0.95) (**Table 3**, Figure 1A). Due to the difference of average body temperature among different groups, ROC analysis was conducted respectively. Remarkably, the accuracy of NCITs varied greatly among different groups of individuals. In children, the highest AUC of body surface sites with NCITs was the neck (0.902) whereas AUC of neck, temple and forehead in teens and adults (female) were 0.981, 0.973 and 0.961 respectively (Figures 1B,C). Among teens and adults (female), the accuracy of wrist temperature was the lowest; the AUC was only 0.887 (Table 2, Supplementary Figures 1A–D).

**Table 2:** Description of diagnostic effectiveness of NCITs on the four surface sites.

**Variables**	**AUC**	**95%CI**	**Cut-off value**	**Sensitivity**	**Specificity**	**Youden index**
Total						
Neck temperature	0.922	(0.901, 0.939)	36.65	0.956	0.623	0.579
Temporal temperature	0.911	(0.892, 0.937)	36.45	0.961	0.617	0.578
Forehead temperature	0.893	(0.863, 0.915)	36.35	0.968	0.398	0.366
Wrist temperature	0.824	(0.797, 0.852)	36.15	0.950	0.343	0.293
Boys						
Neck temperature	0.856	(0.814, 0.898)	36.65	0.953	0.430	0.383
Temporal temperature	0.863	(0.854, 0.905)	36.45	0.969	0.406	0.375
Forehead temperature	0.832	(0.823, 0.903)	36.35	0.974	0.219	0.193
Wrist temperature	0.742	(0.688, 0.795)	36.25	0.969	0.141	0.110
Girls						
Neck temperature	0.905	(0.871, 0.939)	36.55	0.972	0.379	0.351
Temporal temperature	0.883	(0.847, 0.920)	36.35	0.972	0.368	0.340
Forehead temperature	0.855	(0.812, 0.898)	36.35	0.958	0.253	0.211
Wrist temperature	0.826	(0.780, 0.872)	36.15	0.986	0.200	0.186
Teens and adults (female)						
Neck temperature	0.981	(0.969, 0.993)	36.75	0.993	0.858	0.851
Temporal temperature	0.973	(0.956, 0.990)	36.55	0.974	0.874	0.848
Forehead temperature	0.961	(0.936, 0.985)	36.45	0.961	0.783	0.744
Wrist temperature	0.887	(0.841, 0.934)	36.15	0.951	0.434	0.379

AUC, area under curve; CI, confidence interval.

**Figure 2:**
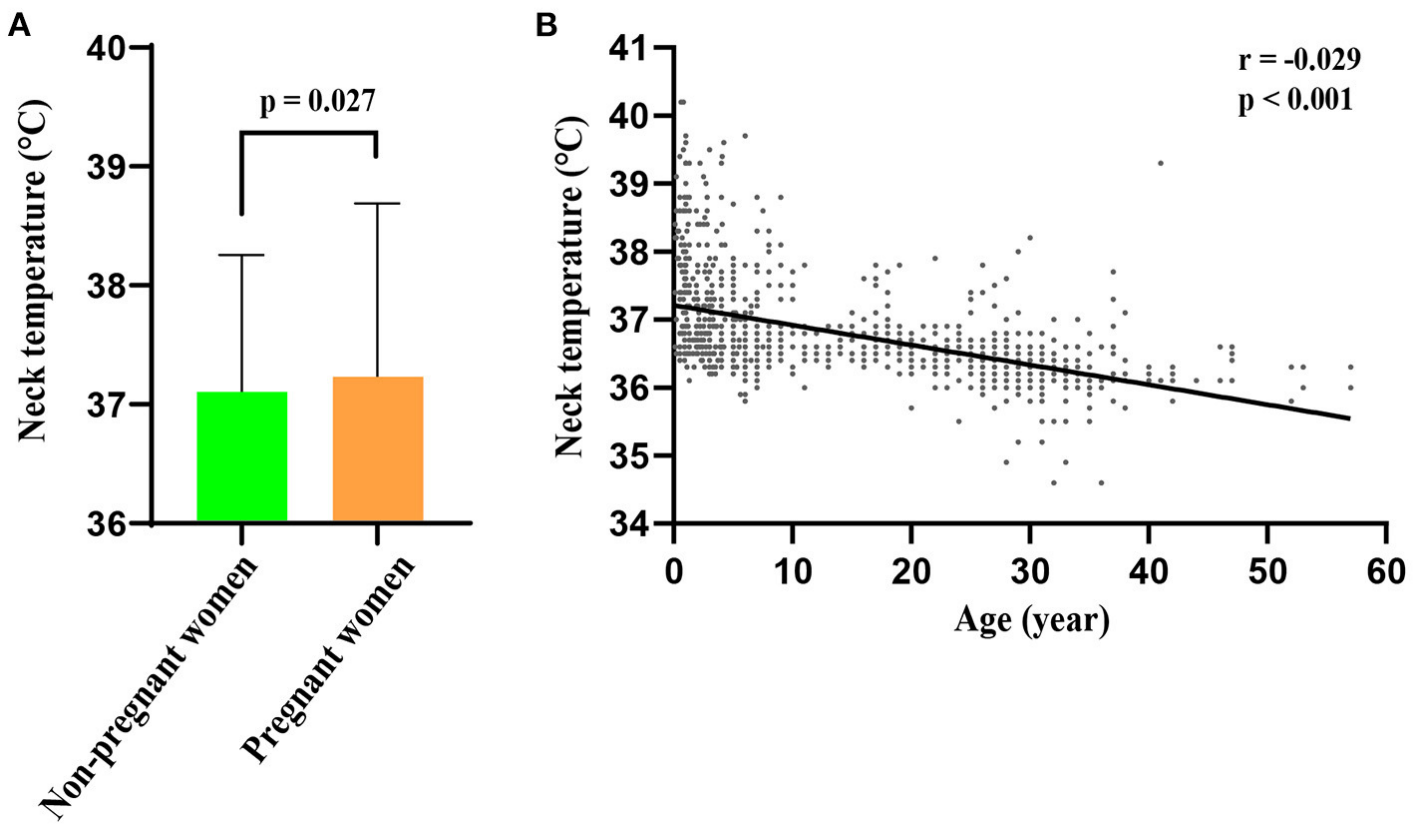
Differences in body temperature among different populations. **(A)** The body temperature of pregnant women was slightly higher than that of non–pregnant women group (*p* = 0.027). **(B)** Linear correlation analysis showed that non–febrile participant ages and their neck temperature were negatively correlated (r = −0.029, *p* < 0.001).

Considering the vast majority of patients with fever are children, and the uncertainty of the accuracy of axillary temperature in children, linear correlation analysis was performed to analyze the correlation between non-febrile participant ages and neck temperature (Figure 2B). Our results revealed that non-febrile participant ages and their neck temperature were negatively correlated (*p* < 0.001). The effect of non-febrile participant covariates on neck temperature was further analyzed using multiple linear regression. Similar to the results of univariate analysis, neck temperature was significantly associated with participants' age and pregnancy (beta-value: 0.372, 95% CI: 0.145, 0.599. *P* = 0.002), but was not found to have a correlation with ambient temperature, sex, investigators, mode of transportation. The neck temperature was negatively correlated with participant's age; neck temperature decreased by 0.03 °C when the participant's age increased by 1 year (beta-value: −0.030, 95% CI: −0.036, −0.024; *P* < 0.001) (Table 3).

**Table 3:** Association between non-febrile participant's covariates and neck temperature.

**Variables**	**Beta**	**95% CI**	***P*–value**	**VIF**
R^2^ = 0.670				
Age (year)	−0.030	(−0.036, −0.024)	<0.001	1.350
Ambient temperature (°C)	0.008	(−0.021, 0.038)	0.582	1.036
Gender (male)	−0.021	(−0.337, 0.295)	0.896	1.328
Pregnancy	0.372	(0.145, 0.599)	0.002	1.025
Investigators	−0.024	(−0.256, 0.208)	0.835	1.039
Mode of transportation	−0.009	(−0.033, 0.014)	0.442	1.167

VIF, variance inflation factor.

The fever diagnostic thresholds at the four body surface measurement sites were listed according to Youden index (Table 4). The optimal fever diagnostic threshold needs to fulfill the requirements of fever screening (single sensitivity > 0.95) and display as high specificity as possible. According to this principle, the optimal diagnostic thresholds of fever at these four body surface sites are 36.75 °C at the neck (Youden index: 0.851, sensitivity: 0.993, specificity: 0.858), 36.55 °C at the temple (Youden index: 0.848, sensitivity: 0.974, specificity: 0.874), 36.45 °C at the forehead (Youden index: 0.744, sensitivity: 0.961, specificity: 0.813), and 36.15 °C at the wrist (Youden index: 0.385, sensitivity: 0.951, specificity: 0.434). Meanwhile, the diagnostic threshold of fever with NCITs at the neck was 36.7–37.8 °C, which all exhibited quite high accuracy (Supplementary Table 2).

**Table 4:** The optimal diagnostic threshold of fever at different body surface sites with NCITs in teens and adults (female).

**Variables**	**Cut–off**	**Sensitivity**	**Specificity**	**Youden**
	**value**			**index**
Neck temperature	36.75	0.993	0.858	0.851
Temporal temperature	36.55	0.974	0.874	0.848
Forehead temperature	36.45	0.961	0.813	0.744
Wrist temperature	36.15	0.951	0.434	0.385

The body temperature detected by NCITs needs to be 5 cm away from the body surface, therefore, the effect of ambient temperature on NCITs was further evaluated (Supplementary Figure 2). When the ambient temperature is below 18 °C, the accuracy of NCITs decreases as ambient temperature decreases. Nevertheless, neck temperature is less affected by ambient temperature., the AUC of neck temperature was still high at 9°C (0.923) and commonly remains above 0.950 when ambient temperature fluctuated between 9–29 °C.

In addition, the participants' preference data on body temperature measurement sites from the standardized questionnaire were counted (Supplementary Table 1). Participants preferred to measure their temperature at their wrists or foreheads, mostly for “convenience” and “speed.” More than half of the participants (54.2%) were indifferent to body temperature measurement sites whereas 457 (25.2%) disliked axillary temperature due to “inconvenience” and “time-consuming.” It is noteworthy that our investigators also disliked axillary temperature and were uncertain about the accuracy of axillary temperature measurement with MATs for children because the children did not cooperate well when having the temperatures taken. Failure of body temperature measurement with MATs on children is common.

## Discussion

This further study collected more fever screening data and attempted to ascertain the optimum diagnostic threshold of fever with NICTs at different body surface measurement sites for improving the standardization and accuracy in fever screening under COVID-19.

Consistent with previous studies, in our study, the average body temperature was inconsistent at different body surface measurement sites and different population groups (33, 34). The average body temperature of children was higher than that of teenagers and adults, and that of pregnant women was slightly higher than that of non-pregnant women. This is obvious because children experience more vigorous physiological metabolism and pregnant women undertake the metabolism of the fetus (35, 36). Meanwhile, other factors affecting body temperature [ovulation cycle and circadian rhythm (37, 38), for example] were not involved in this study. However, the method of fever screening should have universal applicability to meet the screening requirements in various situations. Research based on the real-world practice may also meet the universality of fever screening standards.

In our study, the temperatures at the forehead, temples and wrists measured by NCITs were lower than the axillary temperatures measured by MATs whereas the neck temperature was slightly higher; these results are similar to those of previous studies (30, 39). This is obvious because the forehead and temple are bare and are more likely to be affected by ambient temperature. Meanwhile, the wrist is away from the core of the body, resulting in less heat supply. Hence, the higher accuracy of neck temperature can be explained. NCITs are indeed more affected by ambient temperature than MATs because they are not in direct contact with the skin. Moreover, different body surface sites are differently affected by ambient temperatures. As mentioned above, the forehead and temple sites are more affected due to exposure with the neck wrapped in a collar. Therefore, the accuracy of neck temperature is high under different ambient temperatures.

It is worth mentioning that there are also some factors affecting the measurement results in the use of NCITs, although they have been controlled in this study, such as the distance from the skin, the measurement angle and the ambient light intensity (40, 41). According to the standardized body temperature measurement process, we strictly measured body temperature at a distance of 5 cm perpendicular to the skin in a same semi-enclosed artificial light source measurement environment. The body surface temperature measured at same body surface site with NCITs in different studies could be different, which may fluctuate between 34 and 37 °C (42–45). As we said, the accuracy of NCITs may vary according to ambient temperature, measurement distance, the measurement angle, the ambient light intensity, model and manufacturer, etc., the measurement result even has an error of more than 1°C (41). That's why we strictly measured body temperature at a distance of 5cm perpendicular to the skin in a same semi-enclosed artificial light source measurement environment according to the standardized body temperature measurement process. When NCITs are used for fever screening, they shall be measured perpendicular to the surface in strict accordance with their recommended measurement distance in a suitable artificial light environment to improve the accuracy of the results. Meanwhile, a previous study has shown that the additional monitoring of ambient temperature can be strongly helpful to improve the accuracy in the detection of human body temperature, the measurement of which can be affected by the environmental humidity (46). This means that monitoring environment humidity while measuring body temperature or using instruments that can simultaneously measure body temperature and humidity may also increase the accuracy of body temperature measurement.

From our findings, there seems to be a significant difference in the accuracy of NCITs between children and adults. However, the criterion for the accuracy of NCITs is the axillary temperature measured by MATs. Previous studies revealed that the accuracy of MATs in measuring axillary temperature of children has been questioned (47, 48). Because MATs require subjects to clamp their arms for a long time, it is difficult for active children. Moreover, due to the rapidity of NCITs and the researchers' measurement in strict accordance with the standardized process, the obtained temperature data of NCITs were highly accurate in adults. There is reason to believe that NCITs has the same high accuracy in children, and it may be a more accurate method of child thermometry.

In our previous preliminary studies, we have calculated the theoretical optimal diagnostic threshold (maximal sum of sensitivity and specificity) of each body surface site (27). For example, when 37.4 °C at the neck is the diagnostic threshold of fever, the sensitivity is 0.866 while specificity is 0.846. However, the sensitivity of 0.866 does not meet the criteria for fever screening. In actual fever screening, we tend to reduce the fever threshold to improve the sensitivity. Moreover, in the preliminary study, the inaccuracy of children's axillary temperature data was not taken into account, which may slightly affect the determination of the optimal threshold. In this study, we determined the optimal fever diagnostic thresholds at the four body surface measurement sites when the sensitivity fulfilled the requirements for fever screening (single sensitivity > 0.95). It is noteworthy that wrist temperature does not appear to be suitable for fever screening due to its low accuracy (49–51).

Although this is the third year of the COVID-19 outbreak, COVID-19 is still quite threatening due to the potential for several dangerous variants to evolve. It is therefore crucial to explore further measures for pandemic prevention and control, which may prepare for potential variant of COVID-19 or future other virus pandemics (13). Fever screening is an effective and low-cost method to detect infectors associated with different variants of COVID-19 or future other virus based on the fact that most infectors with virus infection have fever symptoms (11). Determining the optimal diagnostic threshold for fever screening using NICTs at different body surface sites is instrumental in improving the accuracy of fever screening and providing theoretical reference for healthcare policy. Vaccinate before outbreak of the epidemic (52, 56). And in case of sporadic outbreak, the possible infectors can be found as soon as possible through fever screening and nucleic acid detection, so as to avoid the occurrence of pandemic.

This study is large-scale and with highly accurate data attributed to the standardized body temperature measurement process by the same investigators using the same instruments. Compared with the previous NCITs studies whose main participants had normal body temperatures, nearly half of the participants had “fever,” which can provide a more optimal diagnostic threshold of fever.

This study adds to our understanding of the optimal fever diagnostic thresholds of NCITs at the wrist, forehead, temple and neck under COVID-19. However, there are some limitations that should be considered. First, ambient temperature fluctuated between 9 and 32°C during the study period. Therefore, the accuracy of NCITs beyond these ambient temperatures cannot be assumed. Second, although fever screening is an effective and low-cost method to detect infectors associated with different variants of COVID-19 since most infectors with COVID-19 have fever symptoms, fever screening cannot screen out all infected persons. Third, the study only involved body surface sites commonly used in fever screening in China, and other body surface sites deemed to have high accuracy, like the inner ear and canthus (53–55), were not included. Meanwhile, the circadian rhythm and female menstrual cycle may also be influencing factors of body temperature, but this study is conducted in the outpatient and emergency department, so the patient's temperature cannot be measured continuously, the effect of the circadian rhythm and female menstrual cycle on body temperature cannot be analyzed.

## Conclusion

Although our study confirmed the high accuracy of NCITs in fever screening when used correctly, it should be noted that NCITs need to be measured perpendicular to the surface in strict accordance with their recommended measurement distance in a suitable artificial light environment to ensure the accuracy of the results. Given the findings of our study, we recommend 36.15, 36.45, 36.55, and 36.75 °C as the diagnostic thresholds of fever at the wrist, forehead, temple and neck, respectively. Among the four surface sites, neck temperature exhibited the highest accuracy and stability. These results are instrumental in improving the accuracy of fever screening and providing theoretical reference for healthcare policy.

## Data availability statement

The raw data supporting the conclusions of this article will be made available by the authors, without undue reservation.

## Ethics statement

The studies involving human participants were reviewed and approved by Ethics Committee of the Chengdu Women's and Children's Central Hospital (No. 202040). Written informed consent to participate in this study was provided by the participants' legal guardian/next of kin.

## Author contributions

XY, YLin, and YLi contributed to the policy-making and administrative work in this study. FL conceived and designed this study and also joined in the data collection. XL and TL analyzed, interpreted, and visualized the data. XW, QW, SC, SW, YX, QH, XZ, and YY participated in the data collection and analysis. All co-authors contributed to the drafting and revising of this research and showed their approval for the publication of the article. All authors agree to be accountable for the accuracy and integrity of this work.

## Funding

Financial support of this work was provided by Chengdu Science and Technology Bureau (22ZDYF1597, 2021-YF09-00048-SN, 2022-YF05-01326-SN, and 2020-YF05-00145-SN), Chengdu Municipal Health Commission (Nos. 2020216 and 2022441), and Chengdu Women's and Children's Central Hospital (No. 2020JC01).

## Conflict of interest

The authors declare that the research was conducted in the absence of any commercial or financial relationships that could be construed as a potential conflict of interest.

## Publisher's note

All claims expressed in this article are solely those of the authors and do not necessarily represent those of their affiliated organizations, or those of the publisher, the editors and the reviewers. Any product that may be evaluated in this article, or claim that may be made by its manufacturer, is not guaranteed or endorsed by the publisher.

## Supplementary material

Supplementary Figure 1 ROC curves were used to reflect the accuracy of NCITs at different body surface sites in different groups. **(A–D)** When axillary temperature ≥ 37.3 °C was used as the diagnostic criterion of fever, ROC curves of fever in boys, girls, non–pregnant women and pregnant women, respectively were shown.

Supplementary Figure 2 The accuracy of NCITs at the four surface measurement sites at various ambient temperatures. When ambient temperature is lower than 18°C, the accuracy of NCITs decreases with the decrease of ambient temperature. Yet neck temperature is less affected by ambient temperature, the AUC of neck temperature was still high at 9°C (0.923) and commonly above 0.950 when ambient temperature fluctuated between 9 °C ~ 29 °C.

## Abbreviations

CI: confidence interval
NCITs: non-contact infrared thermometers
AUC: area under the curve
MATs: mercury axillary thermometers
ROC: curve receiver operating characteristic curve.
