# Supplementary figures and images for: Optimal diagnostic fever thresholds using non-contact infrared thermometers under COVID-19
Source: Front Public Health. 2022 Nov 24;10:985553. doi: 10.3389/fpubh.2022.985553 (PMC9730337; doi:10.3389/fpubh.2022.985553)

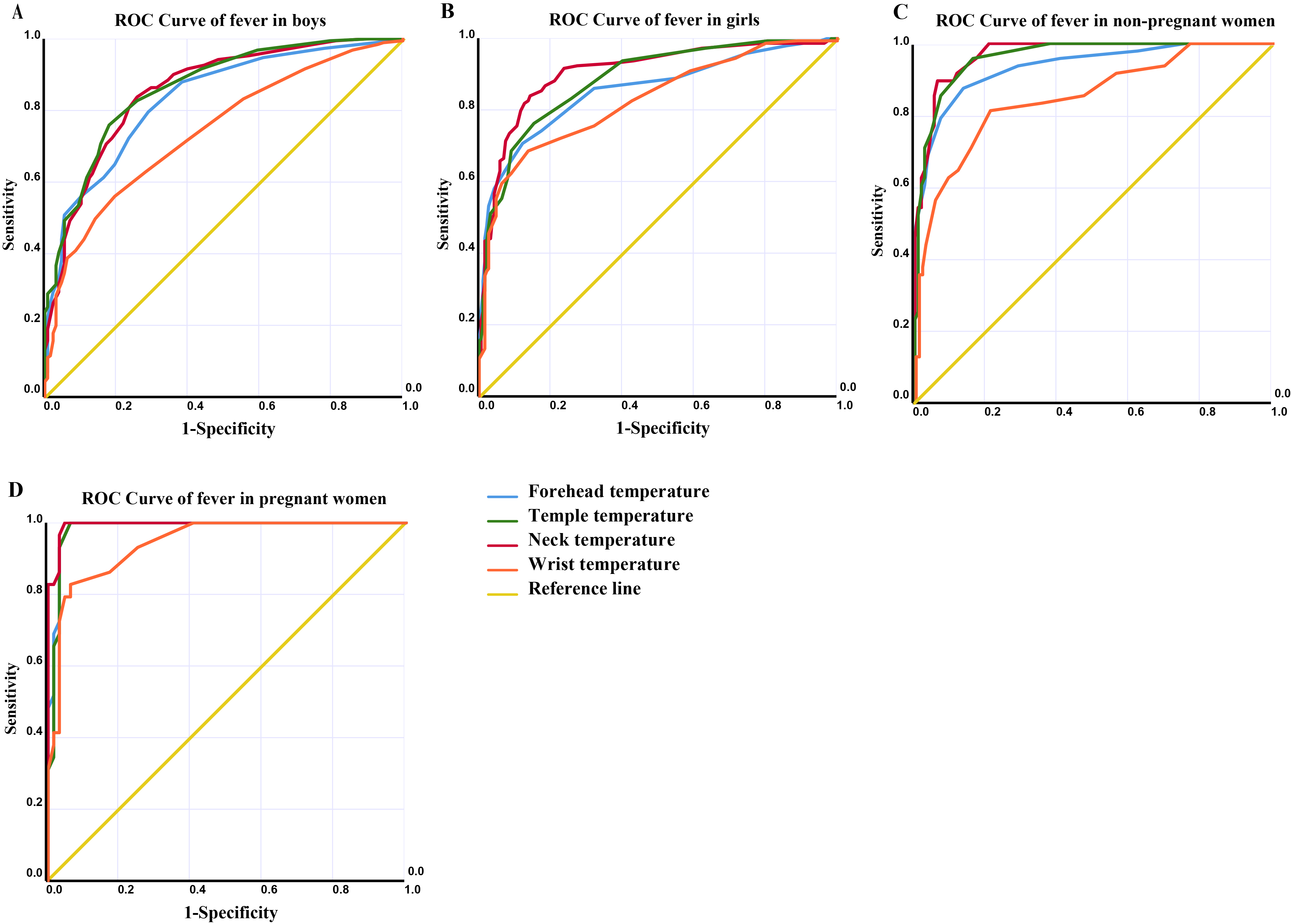

Supplement: Supplementary Figure 1 — ROC curves were used to reflect the accuracy of NCITs at different body surface sites in different groups. (A–D) When axillary temperature ≥ 37.3 °C was used as the diagnostic criterion of fever, ROC curves of fever in boys, girls, non–pregnant women and pregnant women, respectively were shown. [file Image_1.JPEG]

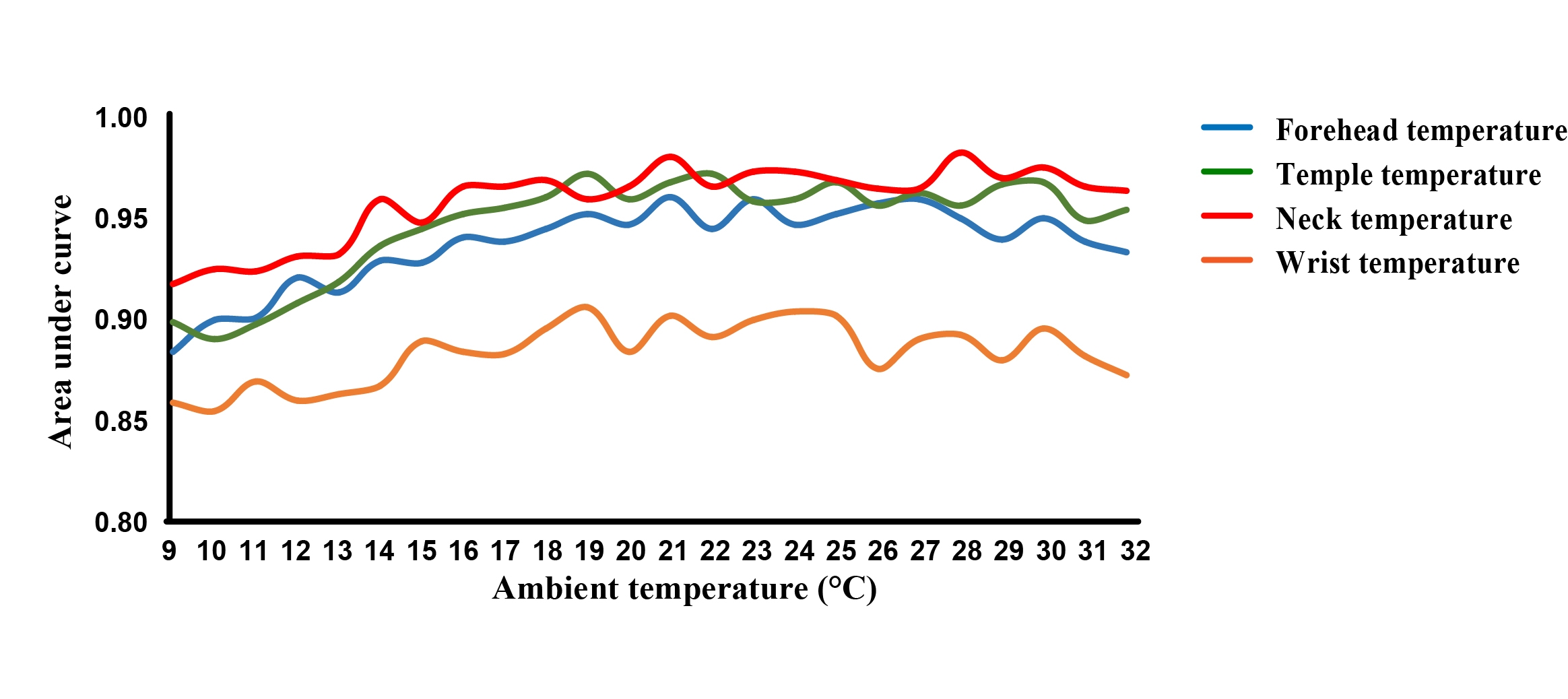

Supplement: Supplementary Figure 2 — The accuracy of NCITs at the four surface measurement sites at various ambient temperatures. When ambient temperature is lower than 18°C, the accuracy of NCITs decreases with the decrease of ambient temperature. Yet neck temperature is less affected by ambient temperature, the AUC of neck temperature was still high at 9°C (0.923) and commonly above 0.950 when ambient temperature fluctuated between 9 °C ~ 29 °C. [file Image_2.JPEG]
