# Supplementary material for: Optimal diagnostic fever thresholds using non-contact infrared thermometers under COVID-19
Source: Front Public Health. 2022 Nov 24;10:985553. doi: 10.3389/fpubh.2022.985553 (PMC9730337; doi:10.3389/fpubh.2022.985553)
Supplement: Supplementary file 3 [file Table_1.docx]

**Supplementary table 1 Evaluation of participants on the five temperature measurement sites**

| Variables | Preference | Dislike |
| --- | --- | --- |
| Forehead temperature | 290(15.6%) | 121(6.7%) |
| Temple temperature | 40(2.2%) | 114(6.3%) |
| Neck temperature | 197(10.6%) | 86(4.7%) |
| Wrist temperature | 451(26.3%) | 55(2.9%) |
| Axillary temperature | 259(13.9%) | 457(25.2%) |
| Indifference | 585(31.4%) | 977(54.2%) |
| Total | 1860(100%) | 1860(100%) |

**Supplementary table 2 The optimal diagnostic threshold of fever at different body surface sites with NCITs in teens and adults (female) (Detailed Edition)**

| Variables | Cut-off value | Sensitivity | Specificity | Youden index |
| --- | --- | --- | --- | --- |
| Neck temperature | 37.45 | 0.922 | 0.949 | 0.871 |
| Neck temperature | 37.35 | 0.922 | 0.937 | 0.859 |
| Neck temperature | 36.75 | 0.993 | 0.858 | 0.851 |
| Neck temperature | 37.55 | 0.896 | 0.954 | 0.850 |
| Neck temperature | 36.95 | 0.935 | 0.914 | 0.849 |
| Neck temperature | 37.15 | 0.922 | 0.926 | 0.848 |
| Temporal temperature | 36.55 | 0.974 | 0.874 | 0.848 |
| Neck temperature | 37.05 | 0.922 | 0.920 | 0.842 |
| Neck temperature | 36.85 | 0.961 | 0.880 | 0.841 |
| Neck temperature | 36.65 | 0.896 | 0.937 | 0.833 |
| Temporal temperature | 36.65 | 0.993 | 0.830 | 0.823 |
| Forehead temperature | 36.55 | 0.922 | 0.891 | 0.813 |
| Neck temperature | 37.65 | 0.844 | 0.954 | 0.798 |
| Forehead temperature | 36.65 | 0.857 | 0.937 | 0.794 |
| Neck temperature | 37.75 | 0.831 | 0.954 | 0.785 |
| ... |  |  |  |  |
| Forehead temperature | 36.45 | 0.961 | 0.813 | 0.744 |
| ... |  |  |  |  |
| Wrist temperature | 36.15 | 0.951 | 0.434 | 0.385 |
