# Supplementary material for: Optimal diagnostic fever thresholds using non-contact infrared thermometers under COVID-19
Source: Front Public Health. 2022 Nov 24;10:985553. doi: 10.3389/fpubh.2022.985553 (PMC9730337; doi:10.3389/fpubh.2022.985553)
Supplement: Supplementary file 4 [file Table_2.DOCX]

# **Questionnaires for the** **NCITs study**

# Participant overview

Participant ID:

Registration date:

Recruitment date:

## Recruitment

### 1. Registeration

* Participant ID： * Participant’s name： *MW Contact Date： *Study ID：

### 2. Agreement

*I agree to take part in this study. Yes NO

1. I have been reading the informed consent, the researchers have explained the purpose, contents, risks and benefits of this research to me clearly. My questions so far have been answered. I understand the information printed on this form, and I volunteered for this study.

Yes NO

1. I agree to use my temperature data for this study. I could withdraw from the study whenever I decide, which won’t affect the normal care in the Chengdu Women's and Children's Central Hospital.

Yes NO

3. I agree the researchers to check on my medical records and personal information related to medicine. I know that my personal information will be kept secret. Yes NO

4. I agreed to the use of personal information I donated in current and future scientific research (including commercial research and scientific research not related to this project). I know clearly that if this research could lead to new treatments and inventions of medical testing, I would not be able to gain commercial benefits from it.

Yes NO

5. I have the opportunity to invite my family or friends to help me to ask questions about this study, I know the person I should contact with if there is a problem. Yes NO

### 3. Enrolment

Inclusion Criteria：* Outpatient and emergency patients

***** Able to provide written, informed consent

Exclusion Criteria: ***** Vital signs were unstable and need to be rescued

***** With mental illness or physical disability/disorder

***** Body surface temperature could not be obtained (such as antipyretic paste covering, etc.)

### 4. Participant’s basic information

*name：

*date of birth：

*native place：

*Telephone number：

* Patient ID：

*education：1- primary school 2- high school 3- University 4- university or above 5- illiterate 6- other:

*occupation：

*blood type：

*smoke（1-no 2- smoking before 3-still smoking, /d 4-History of passive smoking, years） *alcohol（1-no 2- drinking before 3-still drinking, g/week）

### 5. Participants' views

1.(Single choice)Which method of temperature measurement do you prefer？

A.Forehead temperature B. Temple temperature C.Neck temperature

D. Wrist temperature E. Axillary temperature F.Indifference

Reason:

2.(Single choice)Which method of temperature measurement do you dislike？

A.Forehead temperature B. Temple temperature C.Neck temperature

D. Wrist temperature E. Axillary temperature F.Indifference

Reason:
